# The origins of human cumulative culture: from the foraging niche to collective intelligence

**DOI:** 10.1098/rstb.2020.0317

**Published:** 2022-01-31

**Authors:** Andrea Bamberg Migliano, Lucio Vinicius

**Affiliations:** Department of Anthropology, University of Zurich, Zurich, ZH, Switzerland

**Keywords:** cumulative culture, hunter–gatherers, *Homo sapiens*, cultural evolution, hominins

## Abstract

Various studies have investigated cognitive mechanisms underlying culture in humans and other great apes. However, the adaptive reasons for the evolution of uniquely sophisticated cumulative culture in our species remain unclear. We propose that the cultural capabilities of humans are the evolutionary result of a stepwise transition from the ape-like lifestyle of earlier hominins to the foraging niche still observed in extant hunter–gatherers. Recent ethnographic, archaeological and genetic studies have provided compelling evidence that the components of the foraging niche (social egalitarianism, sexual and social division of labour, extensive co-residence and cooperation with unrelated individuals, multilocality, fluid sociality and high between-camp mobility) engendered a unique multilevel social structure where the cognitive mechanisms underlying cultural evolution (high-fidelity transmission, innovation, teaching, recombination, ratcheting) evolved as adaptations. Therefore, multilevel sociality underlies a ‘social ratchet’ or irreversible task specialization splitting the burden of cultural knowledge across individuals, which may explain why human collective intelligence is uniquely able to produce sophisticated cumulative culture. The foraging niche perspective may explain why a complex gene-culture dual inheritance system evolved uniquely in humans and interprets the cultural, morphological and genetic origins of *Homo sapiens* as a process of recombination of innovations appearing in differentiated but interconnected populations.

This article is part of a discussion meeting issue ‘The emergence of collective knowledge and cumulative culture in animals, humans and machines’.

## 1. Background

Human cumulative culture [1–6] differs from the culture in other primates in that it more extensively accumulates over generations without loss, a property described as directional or ‘ratchet’ effect [7]. Human culture extends across multiple minds [8–11] and generally cannot be recreated from scratch [12]. While chimpanzees present cultural traditions and instances of teaching [6,13], evidence of cultural ratcheting beyond three-part tools is so far absent [14]. By contrast, cultural complexity in earlier hominins significantly increased from the earliest stone tools [15] to Late Stone Age and Upper Palaeolithic kits of complex and diversified multipart tools [16].

Various studies have argued that cumulative cultural evolution requires cognitive mechanisms including transmission fidelity [17], innovation [18], teaching [19], shared intentionality [7,20], cultural specialization [21,22] and recombination [23], as well as demographic conditions such as large population size and connectivity [24]. However, why those features only evolved in some hominins remain unknown. A desired shift in perspective from proximate mechanisms to major selective pressures can be achieved by studies of adaptive niche [25–27]. In the context of cumulative culture, this perspective postulates that significant changes in foraging strategies and sociality must have taken place in earlier hominins relative to the niches of extant great apes (box 1). Confirming this expectation, recent experimental and methodological approaches have provided decisive evidence for a link between the unique foraging niche of extant hunter–gatherers and human cumulative culture [10,11,47,48]. In the following, we show how the hunter–gatherer foraging niche provided the adaptive environment for the evolution of cognitive mechanisms and network-based collective intelligence underlying human cumulative culture.

Box 1. **Great ape foraging niches and their cultural implications.**

Cultural traditions have been identified in chimpanzees [14,28] and orangutans [5], and to a lesser extent in bonobos [29] and gorillas [30]. Chimpanzees show the richest cultural diversity with social learning and horizontal transmission of tool traditions [31], vertical transmission along with differentiated matrilines [32], basic teaching between mother and infant [13], a diverse gestural repertoire in dyadic interactions [33] and even between-group cultural transmission [34,35]. Nonetheless, cultural recombination and ratcheting have not been observed beyond three- to fivefold tools or tool-use sequences [6,14,36].

Limits to higher sophistication of cumulative culture in chimpanzees may stem from social features. Related males and unrelated females live in polygynandrous and male-philopatric groups, where males defend large territories [37]. Dominance hierarchies favour more cooperation among males than females or between sexes [38]. Simpler cultural innovations such as moss sponging may be introduced even by juveniles or low-rank individuals [31] and spread horizontally by distant observation. However, complex traits requiring close proximity to tolerant demonstrators may be hindered by dominance and antagonism and are mostly transmitted between mother and infant [39]. Consequently, dominance hierarchies [40] may have a negative effect on cultural exchange between matrilines.

Chimpanzee fission–fusion dynamics provides some opportunities for temporary associations and cultural exchange between juveniles [39]. However, group encounters outside fission–fusion units are often antagonistic [41]. Between-group migration is mostly limited to unrelated females transferring for life, but their initial low rank [42] may limit opportunities for cultural exchange [43]. While bonobos show more tolerance and affiliative between-group interactions, they also exhibit male philopatry and dominance hierarchies with high-ranked females cooperating defensively against males [44], and accordingly tool use is less frequent but still within the chimpanzee range [45]. Thus, while a male chimpanzee may observe tool use in around 20 distinct adult males over a lifetime, the figure is over 300 for hunter–gatherers [46]. In summary, although tool use, complex social learning and learning facilitation between mother and infant probably characterized our last common ancestor with *Pan*, radical changes in adaptive niche had to occur before higher levels of cultural accumulation were possible in the hominin lineage.

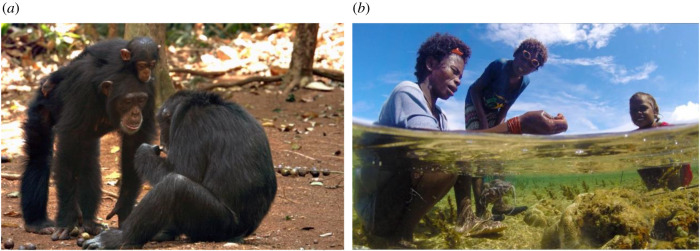

(*a*) Rare case of close-range social learning involving three generations of Bossou female chimpanzees (left). Photo displays the only reported case of a non-emigrating adult female with her mother. Photo credit: Susana Carvalho (Oxford University)/KUPRI (Kyoto University Primate Research Institute). (*b*) Regular behaviour of cooperative foraging with children, and teaching in the Agta hunter–gatherers from the Philippines (right). Photo credit: Rodolph Schlaepfer/University of Zurich.

### (a) Evolution of the human foraging niche: inferences from extant hunter–gatherers

From around 2 Ma, the hominin fossil and archaeological records reveal more persistent stone tool use, occupation of more spatially and temporally variable savannah environments, and exploration of more diverse resources including meat, initially obtained by australopithecines through scavenging and later evolving into high-level scavenging and eventually large-game hunting in *Homo* [36], and underground storage organs [49]. Such subsistence strategy implied larger home ranges and increased mobility compared to australopithecines or extant apes, and dependence on cultural buffering [50]. Those features most likely appeared gradually and were the foundation for the foraging niche of later hunting and gathering hominins including *Homo sapiens*. While it is not possible to reconstruct in full detail the evolution of the foraging niche from its early *Homo* roots (box 2), extant hunter–gatherers can provide invaluable insights into human past adaptations. Different from the fission–fusion groups of chimpanzees and bonobos, hunter–gatherers live in multilevel societies built upon nested levels of organization [86]. Households, household clusters [87], camps and the whole multi-camp structure [11] are recognizable social clusters in hunter–gatherer societies. Clusters interconnectivity is maintained by high rates of inter-camp mobility, with families moving on average every 16 days, with a range between 6 days in the South American Ache and 63 days in the Kalahari Jo/Huansi [88]. Multilevel sociality probably evolved as a consequence of adaptations still observed in hunter–gatherers. While chimpanzees and bonobos are polygynandrous and male philopatric, with cooperation predominantly among related males in chimpanzees [38], monogamy and sex division of labour in hunter–gatherers favour multilocality or dispersal of both sexes, consequently reducing hierarchies within and between sexes [89]. Sex division of labour and biparental provisioning, with consequences such as central place foraging [90], are unique to humans among apes and increase cooperation between sexes and access to resources in multiple camps [91], but also create co-residence with unrelated individuals [92,93] and the challenge of coordinating cooperation among unrelated individuals. The combination of environmental unpredictability and high reproductive costs accounts for food sharing beyond nuclear families [87] and interdependent family units. Extension of cooperative ties and sharing beyond kin may happen owing to shared reproductive interests among in-laws [94], and cooperation among unrelated or even unknown individuals owing to constant mobility and dynamic assortment [88,95]. In conclusion, the fabric of society created by the human foraging niche set the human evolutionary path apart from non-human apes by increasing cooperative ties among kin, affinal kin and unrelated individuals (box 3), as well as promoting specialization, high mobility, fluid sociality and interdependence between family units in open-ended multilevel networks [11,96].

Box 2. **Evolutionary history of the hominin foraging niche.**

Fossil, archaeological and genetic evidence point to a stepwise emergence of the hunter–gatherer foraging niche and cumulative culture in the hominin family. A first transition was observed in some australopithecines and other pre-*Homo* species exploring a wider niche than more specialized hominins [51]. However, while their subsistence style represented an ecological shift favouring increased tool use, there are no indications of associated changes in sociality and therefore no significant improvement in cultural transmission compared to other great apes. Isotope analysis reveals increased reliance on C4 resources in *Australopithecus afarensis* (3.4–2.9 Ma) and *Kenyanthropus platyops* (3.3 Ma) suggesting sporadic scavenging or bone marrow exploration [36,52,53], and possibly tool use for butchering from 3.4 Ma [15,54,55]. However, pronounced sexual dimorphism, higher fractions of non-local strontium isotopes in females and home ranges within the chimpanzee range [56] point to male philopatry with female migration, compatible with steep dominance hierarchies, promiscuous or polygynous mating systems, and no clear departure from ape-like social structuring. Despite the diversity of australopithecine species, their niches were unlikely to provide increased opportunities for social learning, teaching and high-fidelity cultural transmission. Therefore, the emergence of Lomekwian (3.3 Ma) [15] and Oldowan industries (2.6 Ma) [54] in australopithecines may be explained by dietary changes and opportunistic scavenging increasing returns from still occasional tool use [57], rather than by the evolution of a favourable social context for cultural accumulation.

A second niche transition is observed in *Homo* and especially *Homo erectus*, leading to clear changes in sociality and significant facilitation of cultural transmission compared to great apes and australopithecines. The appearance of a more complex tool such as the handaxe suggests the origin of a social environment with increased opportunities for social learning, and the first evidence of possible dependence on teaching and long-term persistence of cultural traditions. Group scavenging [58] in open habitats and shores was associated with increased consumption of meat and aquatic resources near lake shores, evidenced by higher C4/C3 isotope ratios, higher mobility, larger home ranges [53,59] and larger groups inferred both from footprints [60] and comparisons with other primates in open environments [61]. While evidence on sex dimorphism and philopatry is inconclusive, delayed weaning indicated by calcium isotopes [62] suggests provisioning, division of labour and interdependence between sexes. There is also a marked cultural transition in *H. erectus*, with more persistent production of Oldowan tools (from 2 Ma) [57] and the more complex Acheulean tools (1.76 Ma) overlapping in time and sites [63], bone and shell tools [64,65], innovation in handaxe production from 900 ka [66], systematic control of fire from at least 780 ka [67,68] and dispersal routes following raw material sources [69]. In summary, increased within-group cooperation, and possibly gestural teaching [70], may have reduced the risk of cultural loss and facilitated the transmission of technology compared to australopithecines. However, local sourcing of raw materials [71] does not suggest a significant role for between-group exchange, long-range networks or multilevel sociality in *H. erectus*.

A third and most significant shift in foraging niche is noticeable in early *H. sapiens*, with evidence of important changes in social structure and radical consequences for cultural evolution. The foraging style of early humans was characterized by extended ecological ranges, broader diet with specialized large-animal hunting, aquatic exploration and seasonal resource use [16,25]. Those features point to intensified resource and spatial exploration, and resulting changes in social structuring at local and regional scales. For example, there is evidence of larger social networks [72] most likely aided by language and speech [70,73], reuse and structuring of residential sites and presence of family units [16]. Ancient DNA demonstrates changes in group composition in early European hunter–gatherers exhibiting reduced within-group relatedness and inbreeding, suggesting multilocal residence and high inter-group mobility at least 34 ka [72] but possibly much earlier in Africa [74]. Although instances of between-group conflict were identified [75], evidence for cooperative and pacific group interactions between bands are overwhelming. Such changes in social structure and especially the emergence of larger networks at regional scale had profound effects on patterns of cultural transmission, with the increased evidence of cultural recombination and accumulation. Strontium isotope analysis of ostrich eggshell beads from 33 ka exemplifies long-range exchange networks integrating ecologically complementary regions, resembling the exchange of beads in ritual *hxaro* systems of modern Ju'huansi hunter–gatherers [76]. Because *hxaro* beads are currently produced by women, sex division of labour may date back from the Late Stone Age [77]. Transport of obsidian over 160 km [71] and pigments used for artefact and personal decoration at 320 ka [78] hint at a much older origin of regional networks. In stark contrast with the previous hominin record, significant cultural diversity and innovativeness at a regional scale is demonstrated by cultural traditions such as the Aterian (North Africa), Mumbda (East Africa) and Howiesons Port (South Africa) among others [16]. The proliferation of hafted tools from 300 ka [79] and microliths used in composite tools from over 70 ka [80] provide further direct evidence for exuberant cultural recombination and ratcheting. Techniques emerging at separate times during the Middle Stone Age such as pigment and point production at least from 315 ka [78], controlled fire and charcoal from 780 ka [67], and shellfishing from 164 ka [81], were later recombined into ochre-processing kits workshops at 100 ka [82] deploying raw materials from multiple sources. Similar kits were later associated with the first known drawings in South Africa at 75–100 ka [83] and cave paintings in Sulawesi at 44 ka [84], and in the European Upper Palaeolithic at around 35 ka [85]. In summary, the stepwise evolution of the foraging niche has ultimately led to increased substructuring and interconnectivity among human populations, and created the foundations for human cultural accumulation.

Box 3. **Social networking in hunter–gatherers.**

New approaches to the study of mobility and sociality in past and present hunter–gatherers include sensor technologies, remote censoring, image analysis, machine learning, isotope tracking and agent-based simulations among others. Quantification and mapping of hunter–gatherers social networks has revealed details of a fluid and multilevel sociality, where friendship links connect unrelated mobile households into camps of temporary composition, and camps into multi-camp structures [11,96–101]. Friendships start early in playgroups where toddlers already spend more time with unrelated friends than parents [102]. Mobility across networks promotes constant encounters between friends, affinal kin and kin members frequently moving between residential camps [46,94,96]. Between-camp connectivity over long territories buffers against resource unpredictability, seasonality and environmental depletion [91,103]. Ethnographic studies demonstrated the longevity and relevance of friendships to the hunter–gatherer lifestyle [104]. Among the Great Andamanese, a visitor would often adopt the host's child to seal a lifetime bond between unrelated households from different camps [105]. Non-reciprocal adoptions created networks connecting multiple households and camps, with few children residing with their parents.

Long-distance networking is also crucial to foraging, cooperation and cultural exchange. The Kalahari Ju/wa maintain up to 15 *hxaro* friendships, each over up to a 100 mile distant, based on reciprocal exchanges of valuable gifts [106,107], and resulting in exchange networks with hundreds of individuals. Partners are often from different environments and age groups, ensuring diversity in exchanged goods and skills. Around 70% of personal possessions resulted from exchanges, and most visits to distant *xharo* partners had the purpose of exchanging gifts. The Hadza in Tanzania play the *lukuchuko* game, betting for valuable but rare items such as arrowheads, scrap metal, arrow poison or seeds, thus stimulating travelling and spreading of material culture across hundreds of miles [108]. The BaYaka in Central Africa exhibits a system of rituals where spirit guardians demand the sharing of valuable hidden objects, promoting their circulation within and between communities [109]. In summary, traditional ethnography and new quantitative approaches can provide insights into the creation, adaptive functions and cultural consequences of social networks in hunter–gatherers, which rank among the most important social adaptations of humans and are associated with increased collective intelligence and cultural complexity.

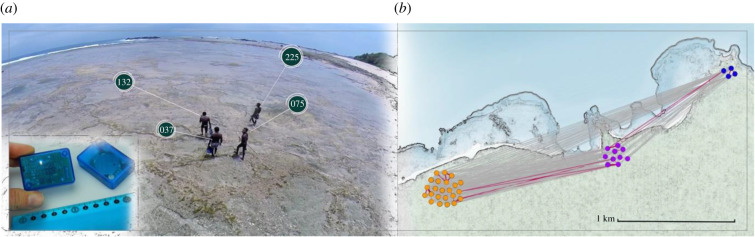

Mapping hunter–gatherer social networks and between-camp migration. New radio sensor technologies ((*a*), insert) can be used to trace contacts between individuals in hunter–gatherer populations (*a*), and reconstruct proximity networks within and between residential camps (dot colours, (*b*)).

### (b) From foraging niche to human cumulative culture

We argue that the foraging niche and its components (pair bonding, multilocality, reduced hierarchies, extended kinship, high mobility, multilevel social networks and prosociality beyond kin) are the explanation for the evolution of human unique cumulative culture. Based on evidence from extant hunter–gatherers, we propose that the foraging niche created the social environment and selective pressures for the evolution of cognitive mechanisms widely recognized as underlying human cumulative culture. Such mechanisms include transmission fidelity, teaching, cultural specialization, recombination and ratcheting. Consequently collective intelligence, generally defined as the increased problem-solving abilities of groups compared to individuals [3,110,111], has been transformed in the human foraging niche. This resulted from the evolution of a ‘network memory’ and ‘social ratcheting’ or irreversible division of labour, spreading the burden of cumulative cultural knowledge across individuals.

### (c) Breakdown of social hierarchies increases fidelity of cultural transmission

We propose that reduction in social hierarchies was a central factor behind the increased efficiency of cultural transmission in humans. In chimpanzees and other non-human primates with dominance hierarchies, reduced social tolerance decreases close physical proximity and opportunities for direct and extended observation of complex cultural behaviours performed by role models [19,112]. Subordinate individuals are also less likely to express learned behaviours or be copied when dominants are present [113,114]. The result is reliance on low-fidelity transmission mechanisms such as emulation [39] or reverse engineering [115]. For example, chimpanzees and bonobos acquire moss-sponging skills by reusing discarded sponges without close contact with skilled users [31]. Reliance on low-fidelity transmission does not seem to result from a lack of ability to imitate or copy actions [116]. For example, wild juvenile chimpanzees imitate infant-caring behaviour using rock-dolls, facilitated by tolerant mothers allowing for close-range observation [117]. We conclude that social hierarchies rather than intrinsic cognitive limitations are the main reason why chimpanzees rely mainly on low-fidelity mechanisms of cultural transmission. We can therefore argue that the reason for increased opportunities for more precise copying in hunter–gatherers [118] is that egalitarianism and social tolerance increase proximity and available time for direct observation of cultural role models differing by age, sex and family [119]. For example, hunter–gatherer children interact freely with unrelated individuals from different age groups and spend the most time in playgroups [102,120], where they imitate each other and practise adult skills by hunting small animals, producing toy tools or simulating rituals based on extended observations of adults [121]. In addition, the density of households in camps and cosleeping of various family members within households reduces physical proximity and enhances social learning [122]. In summary, reduced hierarchies in hunter–gatherers facilitate close-range interactions between learners and role models, providing a social context more favourable to high-fidelity cultural transmission.

### (d) Cooperation between pairs, extended families and households explain teaching in hunter–gatherers

One of the puzzles in the evolution of cumulative culture is the origin of teaching. Although central for cultural transmission and widespread in humans, teaching is very rare in non-human primates. This is generally explained by an unfavourable balance of costs, benefits and relatedness between tutors and pupils [123]. Teaching is predicted to evolve when skills are highly valuable and difficult to acquire, and when tutors are closely related to pupils. This accounts for rare examples of teaching by related helpers, parents or siblings in cooperatively breeding meerkats and callitrichids, eusocial insects, and between mother and offspring dependent on hunting skills (meerkats, cats and cheetahs) [123]. We argue that this is why teaching in chimpanzees has so far only been observed between closely related individuals (mother and offspring) in the context of tool donation and learning facilitation of termite fishing, a complex behaviour bringing significant benefits but hard to learn without social transmission [13]. By contrast, the hunter–gatherer foraging niche has significantly shifted the balance of costs and benefits towards teaching and modulated the effect of relatedness. First, pair bonding spreads the costs of teaching between parents and significantly increases paternal contributions [124,125]. Second, shared reproductive interests further distribute teaching costs and benefits to affinal kin [94]. Finally, cooperative foraging with shared returns allows exploration of high-quality resources among multiple participants, spreading the benefits of teaching unrelated individuals (who can help to increase group production). For example, cooperative octopus hunting by Agta hunter–gatherers provides opportunities for adults to teach youngsters through cooperation and increased returns. We conclude that while teaching may be occasionally observed in other great apes, the social structure of hunter–gatherers including pair bonding, shared reproductive interests and cooperation with unrelated individuals reduces costs and increases the benefits of teaching, facilitating the learning of more complex technologies and social norms.

### (e) Differentiation of network pathways favours cultural specialization within groups

Humans are the only apes where individuals perform highly specialized roles. An explanation for cultural specialization may rest in the unique structure of human social networks and their differentiated interaction channels. For example, in chimpanzees and orangutans, the main channel of cultural transmission and differentiation is transmission across matrilines [32,126]. In hunter–gatherers, social networks are multiple and polyfunctional [127], allowing individuals to interact simultaneously within various specific social groupings differentiated by sex [124], age [128] and skill levels such as female foraging groups, male hunting groups, child playgroups, household units and residential camps. For example, in BaYaka hunter–gatherers medicinal plant knowledge is transmitted between partners, their kin and affinal kin owing to shared reproductive interests, facilitating cultural specialization [10]. Specific transmission channels also stabilize cultural domains and allow for their coexistence and differentiation among social groupings. Restrictions on information flows between channels may further increase cultural diversification [129], as exemplified by BaYaka secret sex-specific rituals [109]. Therefore, we argue that the channelling of information through differentiated interaction paths can explain cultural diversification within hunter–gatherer populations and the emergence of specialized roles such as elephant hunter, honey gatherer, shaman, storyteller, dancer, ritual runner, singer, among others [128,130,131].

### (f) Multilevel sociality accelerates cultural innovation and recombination

Recombination of differentiated tools or techniques is widely proposed as the source of major cultural innovations or technological leaps. Cultural recombination products are easy to observe in contemporary societies [132] and hunter–gatherer groups (as exemplified by the bow and arrow or cocktails of distinct medicinal plants). Recombination is also found in chimpanzees, as in honey gathering requiring the sequential use of three to five tools [14]. However, while Tai chimpanzees exhibit 16 tools with 30 technounits, the Hadza exemplify the simplest tool repertoire among extant hunter–gatherers with 39 tools comprising 92 technounits [14]. Hadza tools are also more complex, with clear evidence of recombination such as arrows using up to nine materials. Furthermore, only humans extensively combine tools and technological traditions to create new techniques, for instance when employing stone pounders to produce plant-based medicines, processing food with pounders and fire [133], or collecting honey with tools and smoke [134]. We argue that the reason for the higher rates of cultural recombination in hunter–gatherers is their multilevel social structuring, which is a network adaptation favouring extensive cultural exchange. Since cultural recombination events are rare and occur in evolutionary time, they are hard to observe in field studies and have been often investigated through simulation studies. A recent simulation of cultural evolution based on real hunter–gatherer social networks showed that observed levels of within-camp connectivity and between-camp mobility can significantly accelerate cultural recombination and major technological leaps [11]. As a result, Agta and BaYaka social networks exhibit ‘small-world’ features displaying both high clustering and reduced low path length, which can explain both cultural specialization among close-knit households, and cultural recombination through friendship links between households and camps [96].

### (g) Network memory promotes cultural complexity and ratcheting

Human cumulative culture is characterized by a ratchet effect, whereby cultural traits survive across generations with relatively little backward slippage and continuous incorporation of innovations [7]. Although non-human primates have provided many examples of long-lasting cultural traditions, the more complex cultural traits of hunter–gatherers suggest higher rates of cultural accumulation. The fact that culture is produced by populations rather than individuals may explain why ratcheting is more efficient in humans. For example, a BaYaka population from Congo demonstrated collective knowledge of 32 medicinal plants, but no individual knew the whole medicinal repertoire [10]. Therefore, the ratchet effect implies reliance on a collective memory that distributes cultural knowledge across individuals unable to fully recreate it from scratch. We argue that a main reason hunter–gatherers build more efficient collective memories is their unique social structure. Simulations have shown that large population size and full network interconnectivity reduce the risk of cultural loss, but also wipe out diversity by homogenizing traits owing to group-wide transmission. On the other hand, fragmented groups may produce more diversity because of differentiation between clusters, but extreme fragmentation may result in isolation, reduced introduction of innovation from other clusters, loss of collective memory in smaller groups and ultimately loss of cultural complexity over time [135,136]. Therefore, the reason for cultural ratcheting in hunter–gatherer societies is that their multilevel social structuring takes advantage of both large population size and fragmentation without their side-effects [96], allowing for innovations to accumulate across generations with reduced cultural loss. By contrast, chimpanzees and bonobos live in more stable and cohesive groups, a social structure accounting for their ability to preserve innovations across generations but also for the relative rarity of major cultural leaps. In summary, multilevel sociality can explain how cultural complexity may evolve owing to a collective or network memory splitting the individual burden of storing more diverse cultural repertoires [11,137–139]. The evolution of a complex ‘network memory’ is therefore a distinguishing feature of a human collective intelligence intrinsically linked to cultural accumulation.

## 2. Discussion: gene-culture coevolution and human evolution

The foraging niche had major evolutionary implications beyond the origins of human cumulative culture. As discussed below, the emergence of culture as a second inheritance system in the hominin lineage has significantly shaped human cognition and evolution.

### (a) Social ratchet and the origins of gene-culture coevolution

It has been shown that culture can relax or increase selection pressures and favour adaptations in various species [140,141]. However, the reasons for the transition from a facultative inheritance system to dependence on a system of gene-culture coevolution in hominins remain a puzzle. We propose that as a result of multiple network channels of cultural differentiation and recombination in the foraging niche, at some point hominin cultural repertoires must have grown to a point where single individuals could not master a significant fraction of accumulated knowledge and techniques, as observed in current hunter–gatherers. The expected irreversible interdependence among specialists was proposed as a feature of major evolutionary transitions [142], with the division of labour and extensive cultural exchange favouring specialization, complementary skills and increased system efficiency [21,27]. Similar to the role played by sexual reproduction in genetic evolution, cultural recombination became the main mechanism generating innovations from a pool of skill-differentiated individuals. While debates have mostly focused on cultural ratcheting, the foraging niche also set in motion a ‘social ratchet’ or trade system where specialization within populations became irreversible. This process generates storytellers and shamans in hunter–gatherers, and later medical doctors and IT specialists in industrial societies, in a process analogous to sexual reproduction and the eventual evolution of interdependent sexes. In summary, the foraging niche has created the behavioural and social conditions for the emergence of social ratcheting, or the cultural specialization and interdependence between specialists. The consequence was the transition from reliance to dependence on culture and hence the process of gene-culture coevolution itself.

### (b) Human cultural cognition was driven by selection for cumulative culture in the foraging niche

Comparative studies have proposed that human cumulative culture is explained by unique cognitive capacities such as theory of mind, teaching, shared goals and intentionality, or a tendency of children to overimitate role models [7]. However, most features have now been identified in other species pointing to a continuum with humans at its higher end, suggesting that variation in cognitive abilities may be the result of differences in intensity of past selective pressures. Therefore, the foraging niche perspective implies that uniquely advanced cognitive abilities in humans evolved as a consequence (or proximate mechanisms) of stronger selection for efficient transmission of cumulative culture (the ultimate or evolutionary cause). For example, the stepwise transition to the foraging niche increased interdependence and reliance on cultural transmission, causing stronger pressure for cooperation and collective problem solving. We propose that the result was the evolution of more sophisticated collective intelligence and shared intentionality [3,7,8,11]. While those cognitive abilities had often been described as causes of human culture from a mechanistic or proximate perspective, they are ultimate consequences of the selective pressures for cumulative culture in the foraging niche. The second consequence of our argument is that language may also have emerged from a cumulative cultural process owing to stronger pressure for efficient cultural transmission. This view is compatible with its possible gestural origin as a tool-making teaching aid [70]. In later hominins with larger and more interconnected social networks, language might have evolved into speech as a more complex communication technology [143,144]. Similarly to stone tool technology, speech-based language is a system of multipart tools (or sentences) built from vocalization units and could therefore have evolved through cultural recombination [145]. In summary, the foraging niche may have provided the selective context for the evolution of cognitive and cultural abilities underlying human cumulative culture.

### (c) The foraging niche accelerated the genetic and cultural evolution of *Homo sapiens*

**Figure 1.**
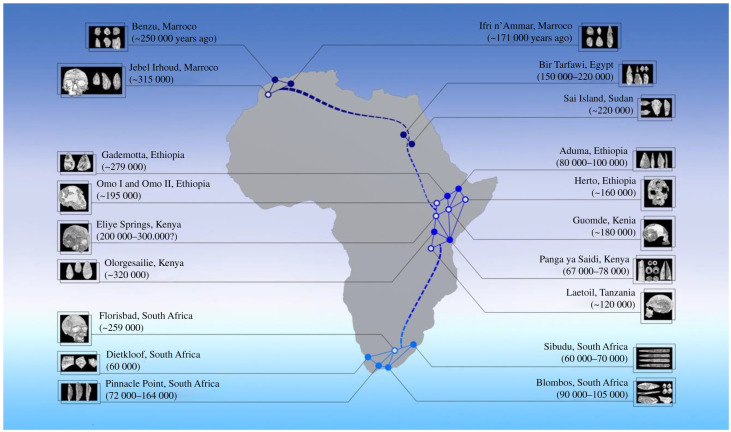
Hypothetical regional networks and the biocultural origins of *Homo sapiens* in Africa. From around 350 ka, the archaeological and fossil records reveal significant diversification of cultural traditions and morphological variation at the continental scale. Three major areas were identified based on local affinities and regional differences in fossil (open circles) and lithic material (solid circles) in north (dark blue), east (blue) and south (light blue) Africa. To explain regional patterns, the figure presents hypothetical large-scale social networks in each region (solid lines) and possible interconnections between regions (dotted lines). Stronger links within regions would account for the regional differentiation of cultural and fossil material, while weaker connections between regions would allow for cultural recombination and genetic exchanges underlying the cultural complexity [82] and morphological differentiation [146] of later *Homo sapiens* populations (see [146–148] for details on fossil and archaeological sites and specimens).

The foraging niche had equally important consequences for the evolution of the human lineage itself. It set some hominin taxa on a path of increasing prosociality, interdependence and cultural exchange dependent on multilevel social structuring, a process reaching its most extreme expression in the larger and fluid metapopulations of early *Homo sapiens*. We propose that large-scale social networks promoted the genetic, morphological and cultural evolution of modern humans by facilitating not only cultural and material exchanges but also flows of people and genes (figure 1). This would explain the accelerated pace of technological evolution in the Middle and Late Stone Ages in Africa [16]. From this perspective, ‘cultural revolutions' such as the Upper Palaeolithic in Europe (possibly incorporating elements from Neanderthal technology [149]) would represent a local case of a continuous process of cultural innovation, recombination and ratcheting within structured hunter–gatherer populations. On the genetic front, large-scale networks may also explain the emergence of modern humans from regionally differentiated early *sapiens* groups identified in east, south and north Africa at 300–400 ka and contributing in different degrees to current modern diversity [146]. The occasional expansion of social networks between species may have also accelerated genetic change in modern humans [150] owing to introgression of adaptive alleles from Neanderthals and Denisovans [151,152]. By the time of Neanderthal extinction in Europe, humans had lived in unrelated and interconnected bands [72] and were the outcome of a long history of cultural and genetic recombination at the continental scale in Africa [25]. By contrast, genetic data indicate that Neanderthals and Denisovans may have faced higher rates of inbreeding [153]. If the latter is an indication of reduced population connectivity, they should also exhibit lower rates of cultural exchange than modern humans. It follows that the adaptive edge of early *sapiens* may have resided in higher cultural recombination levels, as well as superior collective intelligence based on more sophisticated network memories and social ratcheting, rather than differences in individual cognitive ability. Therefore, social and ecological factors may explain why social networks in Neanderthals did not exhibit the levels of regional differentiation and integration observed in *H. sapiens*.

In conclusion, we suggest that a unique foraging niche still observed in a few extant hunter–gatherer populations provided the foundations for human cumulative culture by reducing hierarchies and increasing opportunities for social learning and high-fidelity cultural transmission; facilitating teaching and cooperative skill transfers; promoting sexual and social division of labour and skill specialization; promoting cultural recombination across multilevel social structures; and establishing network memory and social ratcheting processes spreading the burden of cultural knowledge across individuals, resulting in a human collective intelligence uniquely suited to ratcheting culture over generations. The outcome was the eventual transition of the foraging niche into a cultural niche where cumulative culture became a second inheritance system and the main driver of human evolution.

## Data accessibility

This article has no additional data.
